# Poloxamer-Based Thermoreversible Gel for Topical Delivery of Emodin: Influence of P407 and P188 on Solubility of Emodin and Its Application in Cellular Activity Screening

**DOI:** 10.3390/molecules22020246

**Published:** 2017-02-07

**Authors:** Eunmi Ban, Mijung Park, Seonghee Jeong, Taekhyun Kwon, Eun-Hee Kim, Kiwon Jung, Aeri Kim

**Affiliations:** College of Pharmacy, CHA University, 521 CHA Bio Complex, 335 Pangyo-ro, Bundang-gu, Seongnam 463-400, Korea; emban@cha.ac.kr (E.B.); mijungpark826@gmail.com (M.P.); dooblue@hanmail.net (S.J.); chrishyun8@gmail.com (T.K.); ehkim@cha.ac.kr (E.-H.K.); pharmj@cha.ac.kr (K.J.)

**Keywords:** thermoreversible, poloxamer, emodin, solubility, in vitro cellular activity screening

## Abstract

Emodin is a component in a Chinese herb, *Rheum officinale* Baill, traditionally used for diabetes and anticancer. Its poor solubility is one of the major challenges to pharmaceutical scientists. We previously reported on thermoreversible gel formulations based on poloxamer for the topical delivery of emodin. The present study was to understand the effect of poloxamer type on emodin solubility and its application in cellular activity screening. Various gel formulations composed of poloxamer 407 (P407), poloxamer 188 (P188) and PEG400 were prepared and evaluated. Major evaluation parameters were the gelation temperature (Tgel) and solubility of emodin. The emodin solubility increased with increasing poloxamer concentration and the Tgel was modulated by the proper combination of P407. In particular, this study showed that the amount of P407 in thermoreversible poloxamer gel (PG) was the dominant factor in enhancing solubility and P188 was effective at fixing gelation temperature in the desired range. A thermoreversible emodin PG was selected as the proper composition with the liquid state at room temperature and gel state at body temperature. The gel showed the solubility enhancement of emodin at least 100-fold compared to 10% ethanol or water. The thermoreversible formulation was applied for in vitro cellular activity screening in the human dermal fibroblast cell line and DLD-1 colon cancer cell line after dilution with cell culture media. The thermoreversible gel formulation remained as a clear solution in the microplate, which allowed reliable cellular activity screening. In contrast, emodin solution in ethanol or DMSO showed precipitation at the corresponding emodin concentration, complicating data interpretation. In conclusion, the gel formulation is proposed as a useful prototype topical formulation for testing emodin in vivo as well as in vitro.

## 1. Introduction

**Figure 1:**
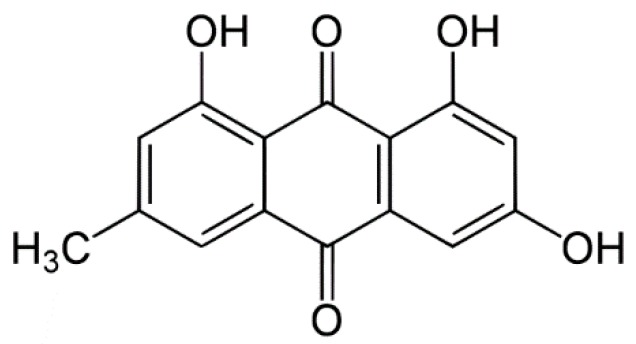
Chemical structure of emodin.

Emodin, 1,3,8-trihydroxy-6-methyl-anthraquinone (Figure 1), is an anthraquinone derivative from the roots of rhubarb (*Rheum officinale* Baill) [1]. Emodin reportedly possesses a number of anti-inflammatory, wound healing and anti-tumor activities [2,3]. Oral bioavailability of emodin is very low because of extensive glucuronidation in liver and intestine [4]. Therefore, the topical application of emodin will be an attractive alternative to oral route for improved bioavailability with less effect by liver or intestinal metabolism. Emodin is known to be soluble in DMSO, ethanol, or alkali solution but insoluble in water. Low aqueous solubility creates obstacles to conducting and interpreting in vivo and in vitro studies for the evaluation of toxicity or pharmacological activity of bioactive molecules, including new drug candidates that have demonstrated clinical effects by several mechanisms. Specifically, during in vitro studies conducted in cell culture media to evaluate biological properties such as efficacy, and genotoxicity, poorly soluble drug candidates might precipitate in the medium, leading to inaccurate information regarding the drug’s properties [5]. Solubility is also a very important factor for performing a functional and pharmacological study of natural compounds because natural compounds such as emodin show activity at concentration of a few tens of μM level. Actually, pharmacological research of emodin has many limitations due to the poor water solubility of emodin although emodin has potential for the treatment of various conditions. Therefore, improvement of emodin solubility is desirable for the evaluation of its pharmacological and functional activity. To date, various approaches to improving solubility have been investigated, including physical or chemical modifications, and the use of solubilizers or surfactants [5,6,7,8]. Among these approaches, the use of a solubilizer or a surfactant is a simple and direct method for improving drug solubility. Poloxamers, composed of various sizes of polymer poly(ethylene oxide) (PEO) and poly(propylene oxide) (PPO) blocks are frequently used with poorly water-soluble compounds, as they are effective surfactants, and lack known toxicity. Poloxamers have also been used in the development of thermoreversible gels for topical drug delivery [9]. Poloxamer 407 (P407) and poloxamer 188 (P188) are the polymers frequently used for preparation of thermoreversible gels and enhancement of drug solubility, respectively. Topical formulations of poloxamer-based thermoreversible gels are being modified to achieve improved compliance and bioavailability [10]. Poloxamer gels (PG) exist as low viscosity solutions at room temperature, but exist as gel at body temperature, making them ideal thermosensitive polymers for topical delivery. Therefore, poloxamer-based thermoreversible gel is a very attractive formulation for topical delivery through the body surface to reduce metabolism and improve the solubility of emodin. Many researchers have studied the effect of varying P407/P188 ratios on properties of thermoreversible gels including gelation temperature [11,12,13,14]. Ban et al. [14] reported the optimization of emodin thermoreversible PG for proper Tgel and emodin solubility by quality by design. In a previous study, we observed that poloxamers are capable of enhancing the water solubility of poorly soluble drugs and P407 is especially more effective than P188 on the enhancement of emodin solubility. In the present study, the influence of P407 and P188 on thermoreversible PG properties and on the solubility of a poorly water-soluble drug was further examined using emodin because less is known about the relative effects of P407 and P188 on drug solubility. Furthermore, the thermoreversible PG formulation was applied for in vitro cellular activity screening in the human dermal fibroblast (HDF) cell line and DLD-1 colon cancer cell line. In our preparations of PG, emodin concentration was constrained at a minimum of 0.5 mg/mL, because previous topical administration studies of emodin held its concentration at approximately 400 μg/mL [15].

## 2. Results and Discussion

### 2.1. Comparison of P407 and P188 for the Solubilization of Emodin

**Figure 2:**
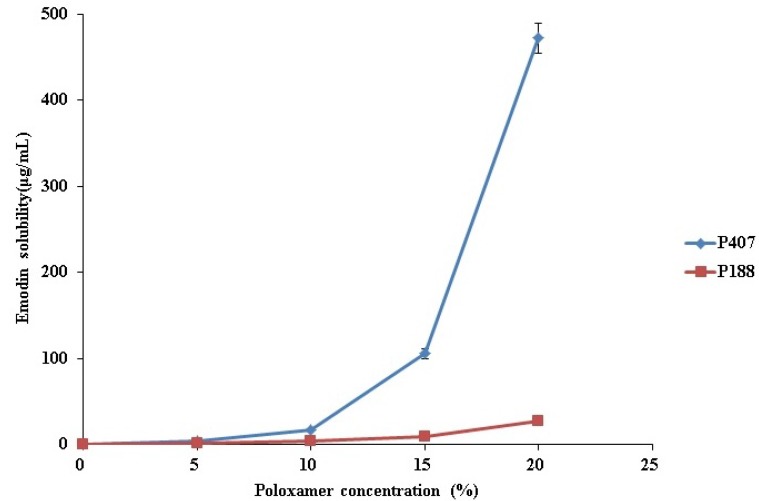
Effect of P407 and 188 concentration on emodin solubility.

In this study, thermoreversible PG containing emodin was prepared using P407 and P188, to determine the individual effects of P188 and P407 on emodin solubility. We measured the solubility of emodin in increasing concentrations of P188 and P407. As shown in Figure 2, emodin solubility increased as the concentrations of both P407 and P188 increased. This result demonstrated that both P407 and P188 have solubilizing properties, and could be used to increase the water solubility of drugs. Of the two, P407 was more effective, particularly when the poloxamer concentration was higher than 10%, due to its higher hydrophobicity and lower critical micelle concentration than P188 (Figure 2). When P407 concentration was 20%, the solubility of emodin was 0.42 mg/mL, which was an appropriate concentration for this study. However, in the case of P188, 40% of P188 was needed to reach 0.5 mg/mL of emodin solubility [14]. Based on these results, we conclude that 20% P407 or 40% P188 was required to solubilize 0.5 mg/mL emodin.

### 2.2. Effect of P407 and P188 on Emodin Solubility and Gelation Temperature in Thermoreversible PG

**Figure 3:**
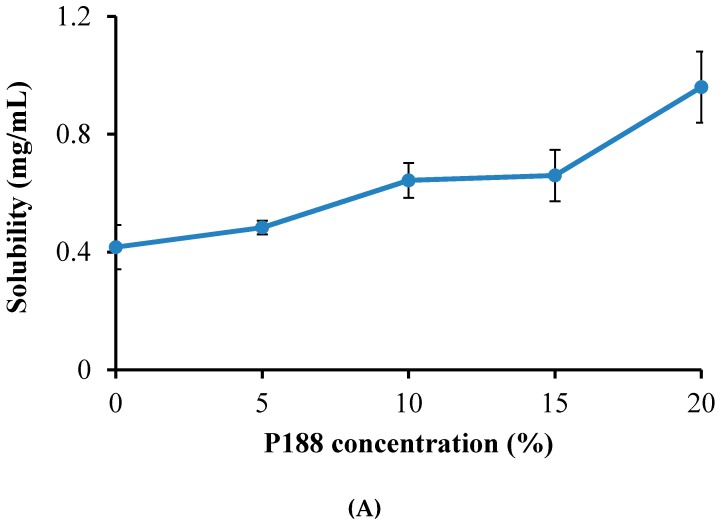

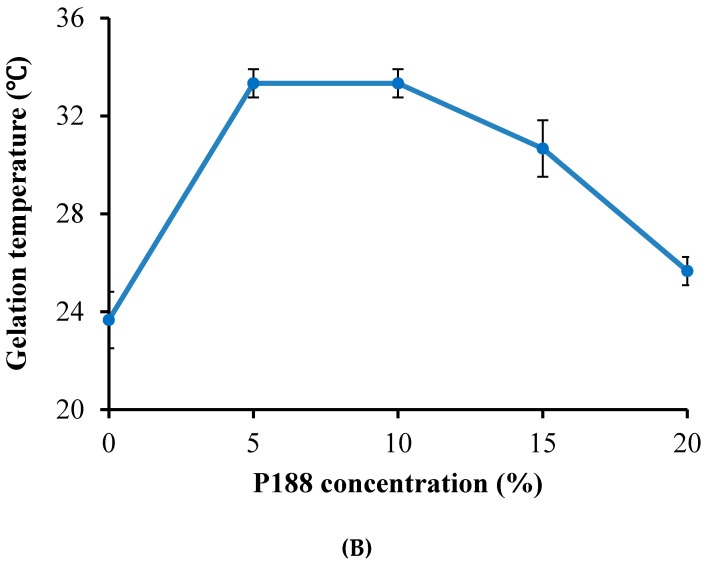
Effect of addition of P188 to the 20% P407 solution on (**A**) the solubility of emodin and (**B**) gelation temperature.

**Figure 4:**
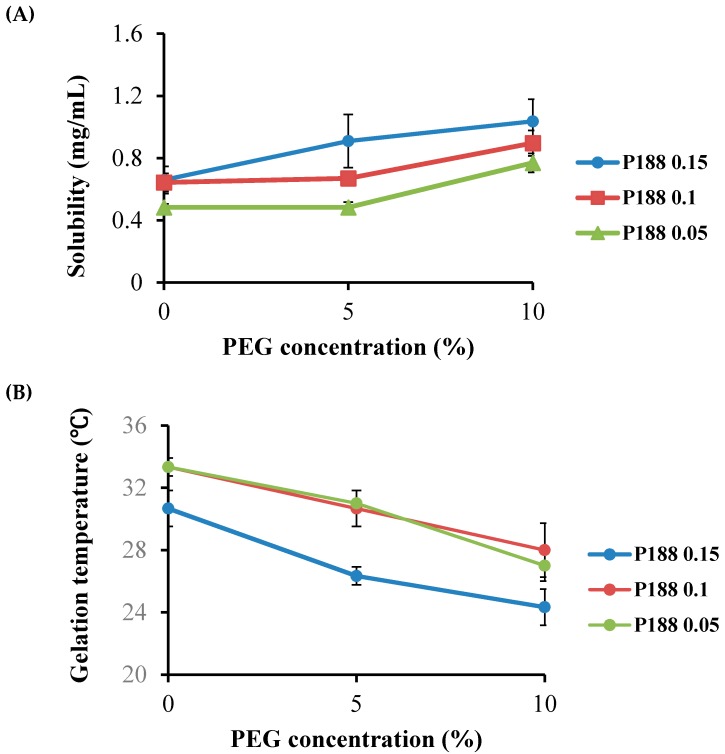
Effect of addition of PEG400 to P188 and P407 mixture at 20% P407 on (**A**) the solubility of emodin and (**B**) gelation temperature.

Thermoreversible PG for the topical delivery of emodin should be gel at skin and body temperature (32–36 °C), while existing as a solution at room temperature. However, PG composed of 20% P407 or 40% P188 solutions did not meet these criteria. The P407 solution gelled below 25 °C, and the 40% P188 solution failed to gel below 50 °C. In this study, two approaches were attempted to obtain PG with the proper gelation temperature and emodin solubility. The first approach was to add P188 to 20% of P407 to increase the gelation temperature with the total poloxamer concentration not greater than 40% (Table 1). In our second approach, varying concentrations of P407 were added to P188 ranging between 20% and 35% with the total poloxamer concentration fixed at 40% (Table 2). It was not feasible to test the total poloxamer concentration greater than 40% because the sample became too viscous to measure emodin solubility. In the first approach, as we expected, the solubility of emodin increased with the addition of P188 to 20% P407 (Figure 3A). The gelation temperature of the PG solution increased from 25 °C to 33 °C with the addition of 5% P188 to the 20% P407 solution. However, when P188 was added to the 20% P407 solution higher than 5%, gelation temperature decreased (Figure 3B), which might be due to shifting of the critical micelle concentration and PEO/PPO ratio at a high concentration of P188. When a larger amount of P188 is added to P407, micellization of P188 begins to predominate [13]. Our results show that PG composed of 20% P407 and 5%–15% P188 had a gelation temperature in the physiological range. Precipitation of emodin in PG during storage at low temperatures, probably due to the temperature-dependent shift in the critical micelle concentration of poloxamer, has been observed. To preserve emodin solubility at low temperatures and retain the desired gelation temperature of PG, various concentrations of PEG400 were added according to the previous study. As shown in Figure 4, addition of PEG400 (5%–10%) to PG resulted in increased solubility but decrease in gelation temperature below 32 °C. Based on these observations, thermosensitive PG composed of P407/P188/PEG400 (20/10/0) was selected considering its gelation temperature in the desired range (32–36 °C) for the topical delivery of emodin (Table 1).

**Figure 5:**
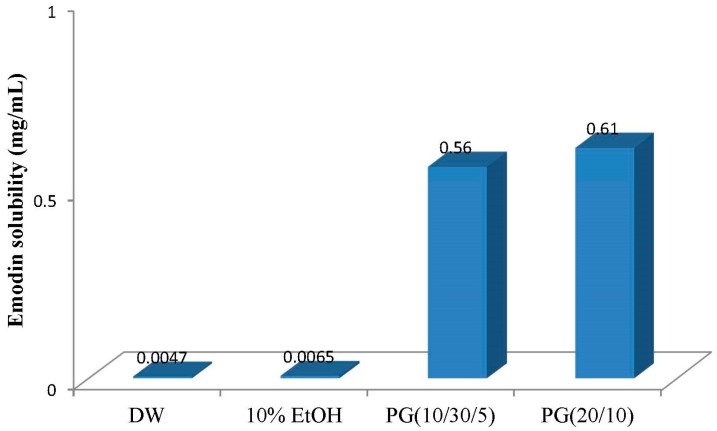
Enhancement of emodin solubility in poloxamer gel.

In our second approach, P407 was added to P188 solution to bring the gelation temperature of PG to within the range near the skin and body temperature (32–36 °C) as in the above study. Varying concentrations of P407 were added to P188 while maintaining a total poloxamer concentration of 40%, and the gelation temperatures were measured at various P407/P188 ratios. In these experiments, emodin solubility increased and gelation temperatures decreased with increasing P407 concentration. PG composed of 5%–10% P407 and 30%–35% P188 had gelation temperatures in the physiological range (Table 2). As in the first approach, PEG400 was added to PG to improve emodin solubility and adjust the gelation temperature (Table 2). The result showed that thermoreversible PG composed of P407/P188/PEG400 (10/30/5) yielded proper gelation temperature for the topical delivery of emodin in the desired range (32–36 °C). The thermoreversible PG with the selected composition enhanced emodin solubility by nearly 100-fold compared to 10% ethanol or water (Figure 5). In addition, of the two thermoreversible PGs, the formulation composed of 20% P407 showed higher emodin solubility than the other although its total poloxamer amount was lower and PEG was not contained as a co-solvent. These results demonstrate that the thermoreversible PG is a useful formulation for solubility enhancement and P407 is a more effective polymer than P188 in terms of solubility.

### 2.3. In Vitro Cell Activity Screening of Emodin Thermoreversible PG

**Figure 6:**
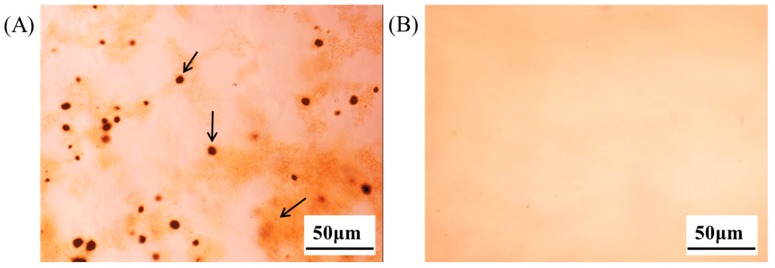
Optical images of clearness of emodin solution diluted with cultured media (scale bar, 50 μm). (**A**) Emodin in ethanol diluted with cultured media and (**B**) emodin in thermoreversible PG diluted with cultured media. Arrows indicate precipitates.

**Figure 7:**
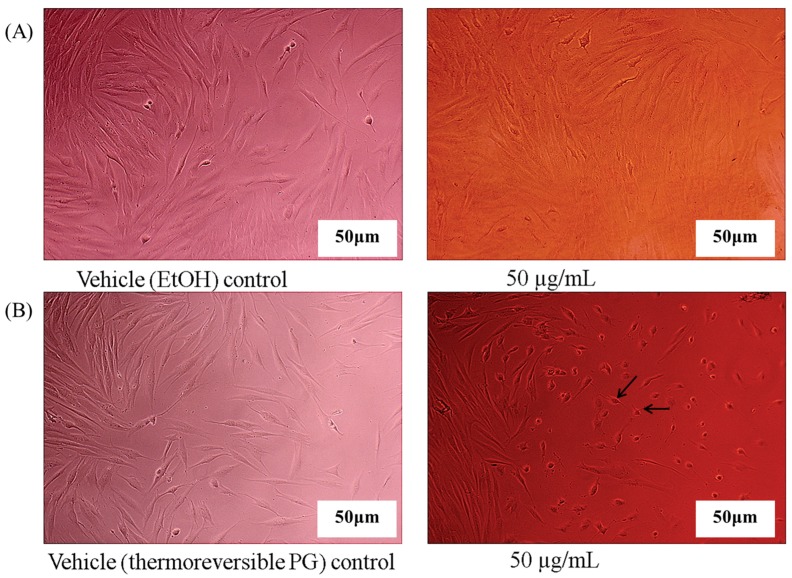
Morphology of human dermal fibroblasts (HDF) cells after treatment of emodin diluted with cultured media (scale bar, 50 μm). (**A**) Morphology of HDF cells after treatment of ethanol (control) and emodin in ethanol diluted with cultured media and (**B**) Morphology of HDF cells after treatment of thermoreversible PG without emodin (vehicle control) and emodin in thermoreversible PG diluted with cultured media. Arrow bars indicate cells with morphology changes.

In vitro cell experiments were performed using thermoreversible PG composed of P407/P188/PEG400 (10/30/5) in order to observe the effect of the enhanced solubility of emodin in thermoreversible PG in vitro cell activity screening. When ethanol solution of emodin was diluted with cell culture media to a concentration of 50 μg/mL, an effective emodin concentration known for various cellular activities, emodin was aggregated and precipitated (arrows in Figure 6A). In contrast, emodin in thermoreversible PG remained as a clear solution after dilution with cell culture media, as shown in Figure 6B. Application of poloxamer to inhibit precipitation was previously reported by Dai et al. [16]. Emodin in ethanol solution of PG diluted with the culture media was treated with HDF or DLD-1 cells to observe effect of emodin on the cell proliferation or cell viability. As shown in Figure 7A, the precipitation of emodin was visually observed and there was no significant difference in cell morphology between emodin and the vehicle control when emodin in ethanol was treated with HDF cells after dilution with the cell culture media. However, when the emodin thermoreversible PG diluted with the cell culture media was treated with HDF cells, the change of HDF cells’ morphology was observed and no precipitation or aggregation of emodin was observed (Figure 7B). The reason that no effect of emodin on HDF was observed after treatment with emodin ethanol solution might be that the amount of cellular uptake of emodin was extremely low because of emodin precipitation and consequently emodin was ineffective at cellular level. In contrast, effective cellular uptake of emodin must have occurred after treatment with emodin in thermoreversible PG due to the enhanced solubility of emodin. We have conducted another in vitro cell study in DLD-1 cells. In this study, emodin was prepared in DMSO and thermoreversible gel, respectively and treated with DLD-1 cells after dilution to various concentrations (5, 25, 50, 100 μM) with the cell culture media. The cell viability changed dose independently when emodin solution prepared in DMSO was treated with the cells. The higher the emodin concentration treated, the more the emodin precipitation in the microplates was observed. These results could be due to the precipitation of emodin upon dilution of DMSO in the culture media. In contrast, the change of cell viability was observed dose-dependently without any precipitation when emodin was prepared in thermoreversible PG (data not shown). These results are consistent with those observed with HDF and imply that the thermoreversible PG is an effective formulation for studying emodin in cell culture and its efficacy in vitro.

## 3. Materials and Methods

### 3.1. Materials

Poloxamer 407 (P407) and 188 (P188) were obtained from BASF Co. (Ludwigshafen, Germany). Acetonitrile, ethanol and methanol were purchased from Honeywell Burdick & Jackson (Muskegon, MI, USA). Polyethylenglycol 400 (PEG400), emodin, and phosphoric acid were purchased from Sigma-Aldrich (St. Louis, MO, USA). All other chemicals were of reagent grade and used without further purification.

### 3.2. Preparation of Emodin Loaded Thermoreversible Poloxamer Gel

Poloxamer gel was prepared according to the previous study [14]. Briefly, P188 and P407 was first dissolved in cold water at room temperature under continuous slow agitation. After the completion of dissolution, emodin dissolved in ethanol was added to the poloxamer solution and then stirred. To make emodin-loaded thermoreversible poloxamer gel, PEG400 was finally added to the previously prepared mixture and gently stirred. Emodin loaded thermoreversible poloxamer gel was maintained at 4 °C prior to use.

### 3.3. Measurement of Gelation Temperature

The gelation temperature (Tg) of emodin loaded thermoreversible PG was measured using the tube invert method as described in the our previous study [14]. Vials containing 1 g of hydrogels were incubated in an ice water bath for at least 20 min, in order to allow the samples to equilibrate thermally to the bath temperature. Vials were transferred from an ice-water bath to a water bath. The bath temperature was then increased stepwise (1 °C/5 min) from 10 °C to 50 °C. At each step, the sample vials were removed from the water bath and were inverted immediately to observe the flow of the sample in the vials. The sample remained at the bottom of the vial after inversion at a certain bath temperature, which was recorded as the gelation temperature. All experiments were performed in triplicate.

### 3.4. Measurement of Solubility

Excess emodin was added to the glass vials containing 1 g of thermoreversible PG and the vials were stirred for 24 h in a cold room (3 ± 2 °C). Each sample was centrifuged at 15,000 rpm, 4 °C for 30 min to spin down the undissolved drug (Labogen 1730R, Labogen, Seoul, South Korea). The supernatant was separated and diluted with ethanol and the mobile phase of high-performance liquid chromatography (HPLC). After filtration through a 0.22 μm filter, the emodin concentration was measured by the Shimadzu UFLC HPLC system coupled with a SPD-M20A PDA detector (Shimadzu, Kyoto, Japan). Chromatographic separation was performed using a Waters RP C8 column (4.6 × 150 mm, 5.0 μm, Waters, New York, NY, USA). The mobile phase consisted of 0.1% phosphoric acid in water and methanol (20:80, *v*/*v*) with the flow rate of 1.0 mL/min and injection volume of 20 μL. The PDA detector was set at 254 nm. The data were analyzed using LC solution software (Shimadzu, Japan). All experiments were performed in triplicate.

### 3.5. Cell Culture Experiments

The preciptaion of two emodin formulations—one in thermoreversible PG and the other in organic solvents—was compared in order to confirm the usefulness of emodin thermoreversible PG for in vitro cell culture assays. The culture medium consisted of Dulbecco Modified Eagle Medium (DMEM) (Invitrogen, Carlsbad, CA, USA), 1% fetal bovine serum (FBS) (Invitrogen, Carlsbad, CA, USA), and 1% penicillin/streptomycin. The first experiment was to test precipitaion after dilution with the culture media. The two formulations were diluted in DMEM to the final emodin concentration of 50 μg/mL and 100 μL of each diluted sample was transferred into a 24-microwell plate. Samples were incubated at 37 °C for 6 h and observed under the light microscope (Nikon Eclipse TS100, Tokyo, Japan). In order to demonstrate the importance of compound solubility for in vitro assay, cells were plated in 24 well plates at a density of 5 × 10^3^ cells/well with DMEM medium and grown for 24 h at 37 °C with 5% CO_2_ and 95% air. Two formulations were diluted with warm DMEM (37 °C) and the diluted samples were treated with the cells at the emodin concentration of 50 μg/mL and incubated for 6 h. Cells were photographed under the microscope (Nikon Eclipse TS100, Tokyo, Japan). For the MTT (3-(4,5-Dimethylthiazol-2-yl)-2,5-Diphenyltetrazolium Bromide) assay, DLD-1 cells (human epithelial colon cancer cell lines, ATCC CCL 221) were cultured in 96-well plates in RPMI 1640 medium supplemented with 10% FBS. After dilution of cold emodin thermoreversible PG and emodin in DMSO with warm media, the diluted emodin samples were treated with the cells (5 × 10^3^) at various emodin concentrations. After incubation for 24 h, cells were washed in phosphate-buffered saline (PBS, pH 7.4), added to 50 μL of the 1 mg/mL MTT solution in sterile PBS, and incubated for 4 h at 37 °C. Then, the plate was centrifugated at 3000 rpm for 10 min, supernatant was carefully removed, and remaining adhered cells were dissolved in 50 μL DMSO. The plate was read at 570 nm using an ELISA reader (VERSAMax, Molecular Devices, VWR, Philadelphia, PA, USA).

## 4. Concluding Remarks

In this study, we have prepared thermoreversible PG containing emodin with high emodin solubility and the gelation temperature suited to physiological use. The emodin thermoreversible PG was applied for in vitro cellular activity screening in the HDF and DLD-1 colon cancer cell line to investigate the vehicle effect. The prepared thermoreversible PG was composed of P407, P188, and PEG400, and was a liquid state at room temperature and a gel state at body temperature. The liquid property of thermoreversible PG at room temperature allows convenient handling and administration for topical delivery. The emodin solubility of thermoreversible PG with an optimized ratio of P407, P188, and PEG400 was improved 100-fold as compared to 10% ethanol or water. This study showed that the amount of P407 in thermoreversible PG was the dominant factor in enhancing the emodin solubility and P188 was effective at fixing gelation temperature in the desired range. In addition, the change of the cell viability and morphology was observed without any emodin precipitation or aggregation after treatment of the cells with emodin in thermoreversible PG, unlike in ethanol or DMSO solution. These results demonstrate that drug solubility and vehicle are very important to in vitro cellular study. Therefore, we suggest that the thermoreversible PG reported in the present study, can be effectively applied to the in vitro cellular study of poorly soluble compounds including emodin.

## Figures and Tables

**Table 1:** Composition and gelation temperature and the emodin solubility of thermoreversible poloxamer gel (PG) based on the poloxamer 407 system.

Poloxamer (%, *w*/*w*)		PEG400 (%, *w*/*w*)	Gelation Temperature (°C)	Solubility (mg/mL)
P407	P188			
20	0	0	23.67 ± 1.15	0.42 ± 0.05
20	5	0	33.33 ± 0.58	0.47 ± 0.00
20	5	5	31.00 ± 0.00	0.50 ± 0.03
20	5	10	27.00 ± 1.00	0.74 ± 0.01
20	10	0	33.33 ± 0.58	0.61 ± 0.01
20	10	5	30.67 ± 1.15	0.66 ± 0.01
20	10	0	28.00 ± 1.73	0.85 ± 0.01
20	15	0	30.67 ± 1.15	0.63 ± 0.01
20	15	5	26.33 ± 0.58	0.85 ± 0.07
20	15	20	24.33 ± 1.15	0.96 ± 0.00
20	20	0	25.67 ± 0.58	0.90 ± 0.00

**Table 2:** Composition and gelation temperature and the emodin solubility of thermoreversible PG based on the poloxamer 188 system.

Poloxamer (%, *w*/*w*)		PEG400 (%, *w*/*w*)	Gelation Temperature (°C)	Solubility (mg/mL)
P407	P188			
10	30	0	36.5 ± 0.6	0.54 ± 0.06
10	30	5	34.5 ± 0.5	0.56 ± 0.07
10	30	10	<25.0	0.69 ± 0.05
5	35	0	38.7 ± 0.4	0.50 ± 0.05
5	35	5	35.5 ± 0.9	0.52 ± 0.06
5	35	10	30.5 ± 0.4	0.61 ± 0.06

## References

[1] Kuo Y.C., Tsai W.J., Meng H.C., Chen W.P., Yang L.Y., Lin C.Y. (2001). Immune responses in human mesangial cells regulated by emodin from Polygonum hypoleucum Ohwi. Life Sci..

[2] Basu S., Ghosh A., Hazra B. (2005). Evaluation of the antibacterial activity of Ventilago madraspatana Gaertn, Rubia cordifolia Linn and Lantana camara Linn: Isolation of emodin and physcion as active antibacterial agents. Phytother. Res..

[3] Hsu S.C., Chung J.G. (2012). Anticancer potential of emodin. Biomedicine.

[4] Liu W., Tang L., Ye L., Cai Z., Xia B., Zhang J., Hu M., Liu Z. (2010). Species and gender differences affect the metabolism of emodin via glucuronidation. AAPS J..

[5] Kawabata Y., Wada K., Nakatani M., Yamada S., Onoue S. (2011). Formulation design for poor water-soluble drugs based on biopharmaceutics classification system: Basic approaches and practical applications. Int. J. Pharm..

[6] Censi R., Martino P.D. (2015). Polymorph Impact on the Bioavailability and Stability of Poorly Soluble Drugs. Molecules.

[7] Rodriguez-Aller M., Guillarme D., Jean-Luc Veuthey J.L., Gurny R. (2015). Strategies for formulating and delivering poorly water-soluble drugs. J. Drug Deliv. Sci. Technol..

[8] Bhat P.A., Dar A.A., Rather G.M. (2008). Solubilization Capabilities of Some Cationic, Anionic, and Nonionic Surfactants toward the Poorly Water-Soluble Antibiotic Drug Erythromycin. J. Chem. Eng. Data.

[9] Kolašinac N., Kyriakos Kachrimanis K., Homšek I., Branka Grujić B., Zorica Ðurić Z., Ibrić S. (2012). Solubility enhancement of desloratadine by solid dispersion in poloxamers. Int. J. Pharm..

[10] Khateb K.A., Ozhmukhametova E.K., Mussin M.N., Seilkhanov S.K., Rakhypbekov T.K., Lau W.M., Khutoryanskiy V.V. (2016). In situ gelling systems based on Pluronic F127/Pluronic F68 formulations for ocular drug delivery. Int. J. Pharm..

[11] Radivojša M., Grabnar I., Grabnar P.A. (2013). Thermoreversible in situ gelling poloxamer-based systems with chitosan nanocomplexes for prolonged subcutaneous delivery of heparin: Design and in vitro evaluation. Eur. J. Pharm. Sci..

[12] Ban E., Kim C.K. (2013). Design and evaluation of ondansetron liquid suppository for the treatment of emesis. Arch. Pharm. Res..

[13] Qi H., Li L., Huang C., Li W., Wu C. (2006). Optimization and physicochemical characterization of thermosensitive poloxamer gel containing puerarin for ophthalmic use. Chem. Pharm. Bull..

[14] Ban E., Jang D.J., Kim S.J., Park M., Kim A. (2016). Optimization of thermoreversible poloxamer gel system using QbD principle. Pharm. Dev. Technol..

[15] Tang T., Yin L., Yang J., Shan G. (2007). Emodin, an anthraquinone derivative from *Rheum officinale* Baill, enhances cutaneous wound healing in rats. Eur. J. Pharmacol..

[16] Dai W.G., Dong L.C., Li S., Deng Z. (2008). Combination of Pluronic/Vitamin E TPGS as a potential inhibitor of drug precipitation. Int. J. Pharm..

